# Chitosan-Based Nano-Embedded Microparticles: Impact of Nanogel Composition on Physicochemical Properties

**DOI:** 10.3390/pharmaceutics9010001

**Published:** 2016-12-22

**Authors:** Paromita Islam, Jorrit J. Water, Adam Bohr, Jukka Rantanen

**Affiliations:** Section for Pharmaceutical Technology and Engineering, Department of Pharmacy, Faculty of Health and Medical Sciences, University of Copenhagen, Universitetsparken 2, 2100 Copenhagen, Denmark; paromitaislam@gmail.com (P.I.); jorritwater@gmail.com (J.J.W.); adam.bohr@sund.ku.dk (A.B.)

**Keywords:** Chitosan, nanogels, nano-embedded microparticles, spray-drying, drug delivery

## Abstract

Chitosan-based nanogels have been widely applied as drug delivery vehicles. Spray-drying of said nanogels allows for the preparation of dry powder nano-embedded microparticles. In this work, chitosan-based nanogels composed of chitosan, alginate, and/or sodium tri-penta phosphate were investigated, particularly with respect to the impact of composition on the resulting physicochemical properties. Different compositions were obtained as nanogels with sizes ranging from 203 to 561 nm. The addition of alginate and exclusion of sodium tri-penta phosphate led to an increase in nanogel size. The nanogels were subsequently spray-dried to form nano-embedded microparticles with trehalose or mannitol as matrix excipient. The microparticles of different composition were mostly spherical with a smooth surface and a mass median aerodynamic diameter of 6–10 µm. Superior redispersibility was observed for microparticles containing amorphous trehalose. This study demonstrates the potential of nano-embedded microparticles for stabilization and delivery of nanogel-based delivery systems.

## 1. Introduction

Nanoparticles are becoming increasingly popular as drug delivery vehicles in the pharmaceutical field for a wide variety of therapeutic indications. Although nanoparticles can be used for diagnostic applications (e.g., contrast agents for medical imaging), drug delivery is the principal medical application of nanoparticles under investigation and clinical use [1,2]. The success of polymer- and lipid-based drug carrier systems has led to several therapeutic products currently available on the market and under clinical investigation [3]. Nanoparticles are generally used to improve the therapeutic effect of active pharmaceutical compounds by improving accumulation in the intended target tissue(s). This leads to higher local concentrations and reduced systemic exposure of the active pharmaceutical, as well as facilitating a controlled release of the compound [4,5,6]. This versatility is further supported by the ability to administer nanoparticles through all the major administration routes including the oral [7,8], transdermal [9,10], pulmonary [11,12], and, predominantly, parenteral route [13,14].

Nanogels are a class of nanoparticles formed by a liquid–liquid phase separation of the soluble nanogel and the bulk solution. Nanogels are formed by covalent or noncovalent crosslinking between its components, thereby expelling water from their inner structure [15]. This method has been widely applied to actively load the therapeutic payload, resulting in higher drug-loading compared to many solid polymeric particles and liposomal systems. Furthermore, the hydrophilic nature and high water content of nanogels have been shown to improve their in vivo retention [16]. Nanogels have been broadly investigated for the delivery of different therapeutic compounds including nucleic acids [17,18], peptides [19], and proteins [20]. Chitosan-based nanogels are possibly the most popular drug delivery vehicle in this class and commonly complexed with sodium tri-penta phosphate (TPP) [21,22]. The hydrophilic nature of nanogels offers manufacturing advantages by eliminating the need for emulsion-based manufacturing methods and organic solvents that are commonly used in the manufacturing of solid polymeric nanoparticles. 

Solution-based manufacturing methods for nanogels result in a final product consisting of a particle suspension. Nanogels in suspension generally have low colloidal stability, as they are prone to aggregation, precipitation, and degradation (by hydrolysis). The limited colloidal stability of nanoparticles in general is a major challenge for commercialization [23], as evident from the number of commercial products which need to be redispersed prior to administration [24,25,26]. Nanoparticles produced by solution-based manufacturing methods can be processed into dry powder products using a post-manufacturing drying step via methods such as spray-drying and freeze-drying. For nanoparticles of a solid nature, a dry powder may be obtained by removal of the aqueous phase without addition of excipients. However, due to the “physical nature” of nanogels, simple removal of the aqueous phase often leads to irreversible aggregation; therefore, these nanoparticles have been dried in the presence of matrix excipients. Spray-drying of nanogels in the presence of such excipients can entrap the nanogels in a solid matrix of microscale particles, also known as of nano-embedded microparticles (NEMs) [27]. NEMs have been shown to limit the mobility and stabilize the nanogels in a dry state that allows for subsequent redispersion of the nanogels in an aqueous phase [27], effectively stabilizing the nanogels and enabling long-term storage. In addition, the matrix excipient could be used to control the release of the nanogel by using pH-sensitive excipients, further functionalizing the NEMs [28].

Chitosan-based nanogels have previously been formulated into NEMs and reported in limited numbers in the literature for pulmonary delivery purposes [27]. These studies formulated chitosan nanogels for delivery of insulin [29] and ciprofloxacin [30] using lactose and mannitol as the carrier matrix. In this work, a novel chitosan-based nanogel NEMs drug delivery system was prepared and characterized. More specifically, the influence of nanogel composition on the final NEM properties, as well as the influence of choice of NEM matrix excipient, was investigated. Nanogels were prepared by the bulk mixing method and were composed of chitosan, alginate, and/or TPP. The nanogels were subsequently spray-dried in a mannitol or trehalose carrier, after which the physicochemical properties and redispersion of the nanogels were investigated.

## 2. Materials and Methods

### 2.1. Materials

Low-molecular-weight chitosan, TPP (MW: 367.864 g/mol) and medium-molecular-weight sodium alginate (viscosity 200–800 cP, 1 wt % in 1% acetic acid, 25 °C) were purchased from Sigma-Aldrich (St. Louis, MO, USA). Trehalose dihydrate and mannitol were purchased from VWR International (Radnor, PA, USA). The water used in this study was of ultrapure quality. All other chemicals and solvents were of analytical grade and were used without further purification.

### 2.2. Nanogel Preparation

Chitosan nanogels of four different compositions were prepared via bulk mixing. In short, chitosan was dissolved in 1% (*w*/*v*) acetic acid to a final concentration of 0.5–1 mg/mL and depending on the nanogel composition (Table 1), TPP, alginate, or a mixture of TPP + alginate (ratio 4:1, *w*/*w*) was pipetted into the chitosan solution under vortexing, at a 3:1 ratio (*w*/*w*). The final pH of all nanogel suspensions was pH 3.2.

### 2.3. Nanogels Size and Zeta Potential Measurements

The hydrodynamic diameter, polydispersity index (PDI), and zeta potential were determined by dynamic light scattering (DLS) using the Zetasizer Nano ZS (Malvern Instruments, Worcestershire, UK) and analyzed using Malvern v7.02 software (Malvern Instruments). All measurements were performed in triplicates at 25 °C, λ = 633 nm, and 173° scattering angle. Prior to measurement, samples were degassed for 5 min using a Tabletop Ultrasound Cleaner 2510 (Branson Ultrasonic, Danbury, CT, USA). Performance of the equipment was regularly validated using size and zeta potential standards.

### 2.4. NEMs Preparation by Spray-Drying

Nanogels were mixed with aqueous solutions of trehalose or mannitol via mild shaking in order to achieve excipient:nanogel ratios of 10:1 to 20:1 (*w*/*w*). The final suspension concentrations ranged from 3.17 to 6.05 mg/mL and were subsequently spray-dried using a Büchi B-290 spray dryer (Flawil, Switzerland) equipped with a Büchi B-267 dehumidifier. The spray-drying parameters were varied with respect to feedstock composition, feed rate, and inlet and outlet temperature (Table 2). The drying air flow rate and atomizing air flow rate were kept constant at 22.5 m^3^/h and 450 L/h, respectively. The spray-dried powders were collected and stored in a desiccator at room temperature.

### 2.5. Aerodynamic Diameter of NEMs

The aerodynamic particle size of the NEMs was determined in triplicates using an Aerodynamic Particle Size (APS) Spectrometer^®^ 3321 equipped with a Small-Scale Powder Disperser^®^ (TSI, Shoreview, MN, USA) by continuous measurement (20 s). The particle size distribution was converted to mass median aerodynamic diameter (MMAD) using the Aerosol Instrument Manager^®^ software (TSI). 

### 2.6. Scanning Electron Microscopy

The geometric mean diameter and morphology of the NEMs was determined by scanning electron microscopy (SEM) using a Philips XL FEG 30 Scanning Microscope (Amsterdam, The Netherlands) operated at an accelerating voltage of 2 kV. NEMs were applied to stubs covered with double-adhesive carbon tape and sputter-coated (5 nm gold) using an E5200 Auto Sputter Coater (Bio-Rad, Herts, UK). Five representative images of each sample were analyzed at different magnification. From the obtained images, geometric mean diameter of the particles was determined using the Fiji scientific image processing software [31] by measuring the diameter of 200 randomly selected particles from each image.

### 2.7. Transmission Electron Microscopy

The nanogels and redispersed NEM powders (in 1% acetic acid) were examined in a Philips CM100 (Philips, Amsterdam, The Netherlands) transmission electron microscope (TEM) operated at an accelerating voltage of 80 kV. Samples placed on an electron microscopy grid were immersed in phosphotungstic acid solution (negative stain) and blotted with a layer of carbon. After drying, the samples were subsequently imaged using an OSIS Veleta digital slow-scan charge-coupled device (CCD) camera (Olympus Soft Imaging Solutions, Munster, Germany) and processed with the Analysis iTEM software package (Olympus Soft Imaging Solutions). 

### 2.8. X-ray Powder Diffraction (XRPD)

Wide-angle XRPD patterns were recorded on a X’Pert PRO X-ray diffractometer (PANalytical, Almelo, The Netherlands). Samples were measured in Bragg Brentano reflection mode in the 2θ range of 5°–37° using a PIXcel detector (step size of 0.039°). The X-ray source was Ni-filtered Cu K_α1_ radiation (λ = 1.541 Å). The operating current and voltage were 40 mA and 45 kV, respectively. The aluminum sample holder was spun throughout data collection to avoid orientation artifacts.

### 2.9. Thermogravimetric Analysis

Thermogravimetric analysis (TGA) of the NEM powder samples was performed using a Discovery TGA instrument (TA instruments, New Castle, Delaware, USA). Samples were heated from ambient temperature (20 °C) to 300 °C at a heating rate of 10 °C per minute in open aluminum pans.

### 2.10. Statistics 

All experiments/measurements were carried out in triplicate and values are presented as the mean ± standard deviation (SD) unless otherwise stated. To identify statistically significant differences, an unpaired *t*-test analysis was performed. Probability values of *p* < 0.05 were considered significant.

## 3. Results

### 3.1. Nanogel Size Varied Depending on Composition and Concentration

**Figure 1:**
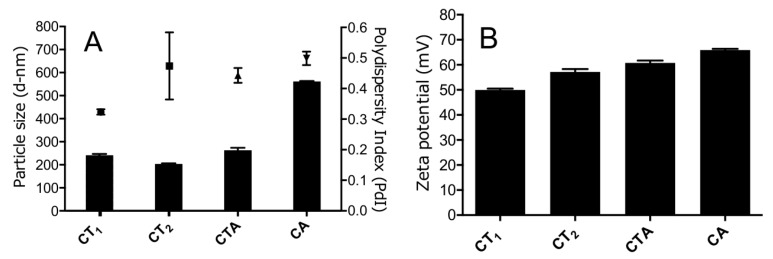
Particle size and zeta potential of the nanogels (mean ± SD, *n* = 3). (**A**) Particle size (bars) and polydispersity index (PDI) (squares); (**B**) zeta potential.

All four nanogel compositions were prepared by bulk mixing and their size and surface charge were subsequently determined (Figure 1). The average hydrodynamic diameters of chitosan–TPP nanogel compositions CT_1_ and CT_2_ were 241 ± 7 and 203 ± 4 nm, respectively. However, nanogel size significantly increased (*p* < 0.001) when alginate was used instead of TPP (CA) at the same weight ratio compared to chitosan (561 ± 3 nm). Addition of TPP to the chitosan and alginate composition (CTA) resulted in a reduced size of 263 ± 15 nm. 

All nanogel compositions had a PDI varying from 0.3 to 0.5, indicating a relatively broad particle size distribution. Increased concentrations of chitosan and TPP during the preparation resulted in a reduction of the PDI as evident from the CT_1_ and CT_2_ compositions. In the compositions containing alginate, the addition of TPP did not have a pronounced effect on the PDI and resulted in a minor PDI decrease from 0.5 to 0.43. 

The zeta potential of the nanogels was positive for all compositions, as expected, due to the cationic charge contribution of chitosan, which was added in excess as compared to the anionic components (alginate and TPP). The CT_1_ composition had the lowest zeta potential value (+50 mV) followed by the same composition at higher absolute concentration (CT_2_). The composition with alginate had the highest zeta potential at +66 mV, while addition of TPP (CTA) resulted in a reduction of the zeta potential. 

**Figure 2:**
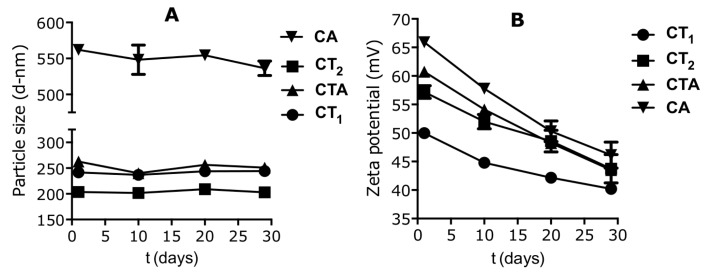
Nanogel size and zeta potential stability (mean ± SD, *n* = 3). (**A**) Nanogel size; (**B**) nanogel zeta potential.

The stability of the nanogels was assessed over a 30-day period after preparation (Figure 2). No major changes in the particle size distributions were observed for all compositions, in contrast to the zeta potential, for which a significant decrease (*p* < 0.01) was observed. All compositions showed a decrease in zeta potential in excess of 10 mV, with the highest reduction (20 mV) observed for the chitosan and alginate composition (CA). 

### 3.2. Influence of Inlet Temperature on the Redispersibility of NEMs

**Figure 3:**
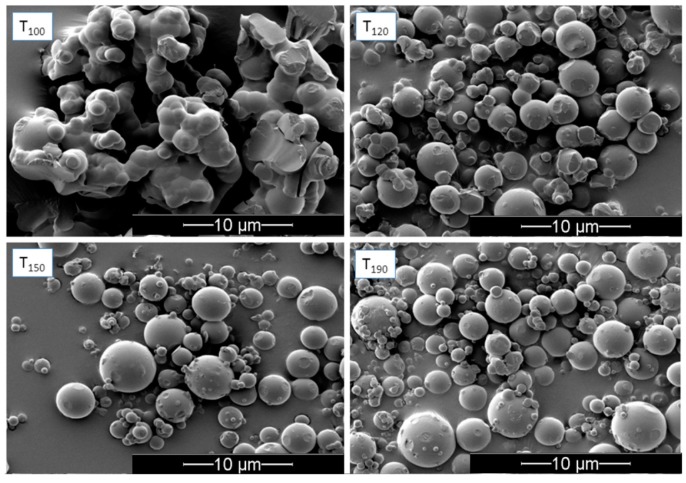
Scanning electron microscopy (SEM) images of trehalose-based NEMs (CTA) spray-dried at indicated inlet temperatures (°C), scale bar: 10 μm.

**Figure 4:**
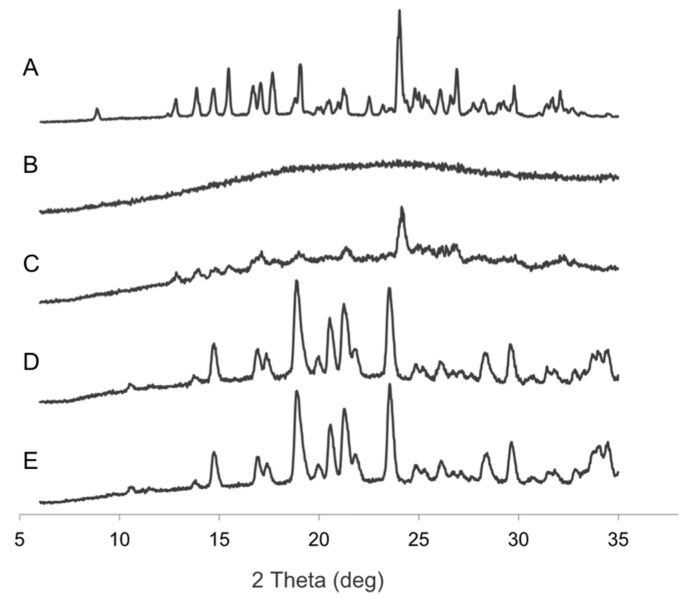
X-ray powder diffraction (XRPD) patterns of unprocessed trehalose dihydrate (**A**); trehalose-based NEMs (CTA) immediately after spray-drying (**B**); trehalose-based NEMs (CTA) two days after spray drying (**C**); unprocessed mannitol (**D**); and mannitol-based NEMs (CTA) (**E**).

In an initial experiment, nanogels of the CTA composition were freshly prepared to investigate the effect of the spray-drying inlet temperature on the resulting physicochemical properties and redispersibility of the NEMs (trehalose-based). This was done to determine a suitable inlet temperature for subsequent experiments. The resulting NEMs were observed as spherical particles in SEM (Figure 3) with an MMAD of approximately 8 μm (Table 2) for each of the selected inlet temperatures (100, 120, 150, and 190 °C). All samples were relatively polydisperse and showed some degree of agglomeration. NEMs prepared at low inlet temperature (100 °C) appeared to be the most agglomerated—mainly via solid bridges—indicating incomplete drying and formation of, first, liquid bridges during the early phase of the spray-drying process, followed by their solidification. This was not directly evident from the TGA measurement, as the moisture content of the NEM powders produced at different inlet temperatures was not significantly different. This is likely due to rapid partial recrystallization of the trehalose in the dihydrate form after sample collection, as confirmed by X-ray powder diffraction (Figure 4). The XRPD analysis indicated that the unprocessed trehalose (starting material) was present mainly in a crystalline state (dihydrate form), which became amorphous upon spray-drying and partly recrystallized within two days after spray-drying, whereas mannitol was crystalline both before and after spray-drying. Any indication of solid form transformation could be observed when comparing the starting materials and NEM powders. SEM images further indicated that samples prepared at 120–190 °C inlet temperature did not form solid bridges, suggesting better redispersion compared with those prepared at 100 °C inlet temperature. The NEMs were subsequently redispersed in an aqueous solution to assess the ability to successfully rehydrate and reconstitute the nanogels from the NEM matrix. These results indicate that an inlet temperature of 120 °C was optimal to obtain redispersed nanogels closest to their original size (i.e., 236 ± 0.09 nm compared to 233 ± 0.04 nm before spray-drying). Both lower and higher inlet temperatures resulted in rehydrated nanogels >300 nm in size. 

### 3.3. Nanogel Composition and Matrix Excipient Impacted Redispersibility

**Figure 5:**
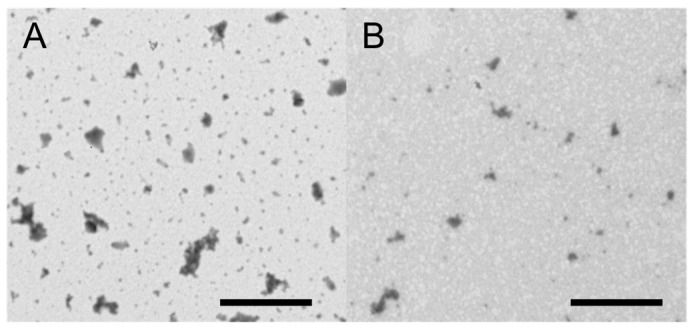
Transmission electron microscopy (TEM) images of nanogels (CTA) composed of chitosan, alginate, and TPP (scale bar: 1 μm). (**A**) Nanogels before spray-drying; (**B**) redispersed nanogels after spray-drying (trehalose matrix).

The other NEM compositions were subsequently prepared with an inlet temperature of 120 °C using trehalose or mannitol as the matrix excipient. The MMAD of the resulting NEMs was dependent on the matrix excipient used as well as the nanogel composition. NEMs prepared with mannitol (Table 3) had an MMAD between 6.68 and 8.23 µm with low residual moisture content of 3–4 wt %, depending on the nanogel composition. As evident from the agglomeration observed in the size measurements, none of the compositions could be successfully redispersed when mannitol was used as the matrix component. Trehalose performed much better as a matrix component, and all compositions were redispersible after spray-drying (Table 4). However, a substantial increase in the nanogel particle size was observed upon redispersion for all compositions. Nanogels composed of chitosan, TPP and alginate (CTA) showed the lowest increase in particle size (≈40 nm) while for the other compositions, a size increase exceeding 170 nm was observed. TEM images (Figure 5) verified the successful redispersion, showing individual nanogels for the redispersed NEMs. The MMADs of NEMs using trehalose as the matrix excipient were similar to those composed with mannitol, with the exception of CT_2_, which were larger at 10.3 µm. This could be due to a concentration-dependent effect, as the CT_1_ formulation and other trehalose-based formulations were all smaller. The moisture content of the trehalose-based NEMs was higher compared to mannitol, between 6% and 8%. Only the NEMs containing nanogels without TPP (sample CA) showed substantially higher moisture content of approximately 12%.

## 4. Discussion

The application of chitosan nanogels for a variety of drug delivery purposes is widespread, and several products based on chitosan nanogels are currently in clinical trials [32,33]. Many of these products in development consist of drug-loaded chitosan nanogels, which are kept as suspensions until administration. However, several studies have indicated that these nanogels are metastable systems, which questions their colloidal stability in a liquid suspension [34]. Furthermore, when loaded with pharmaceutical actives they often suffer from premature release from the nanoparticles, which is difficult to prevent [35]. The application of NEMs to stabilize these systems is therefore of relevance to improve the stability and release profile, and may provide increased functionality in their administration via various dosage forms. NEMs have indeed been shown to functionalize and stabilize nanoparticles, however, little research has investigated the potential of chitosan nanogels for NEM formulation. In addition, not much is known about the influence of different nanogel compositions in NEMs, although a limited number of studies have demonstrated the preparation of NEMs composed with chitosan-based nanogels [29,30,36,37,38].

In this study, chitosan-based nanogels were successfully produced for all of the selected compositions by ionic gelation using a bulk mixing method. As shown, nanogels composed of chitosan and alginate without TPP (CA) were greater in size (561 nm) as compared to the compositions that contained TPP (CT_1_, CT_2_, CTA). The nanogels were, however, stable over a 30-day period. The observed size increase is likely due to the less efficient condensation of the nanogels upon formation, as the alginate is a large “bulkier” polymer as compared to TPP. This would lead to a high degree of entanglement and less efficient packing of the polymer chains, resulting in less expulsion of water from the inner structure. Addition of TPP to the composition reversed this effect and improved the condensation due to the TPP being a small molecule with high anionic charge density, thereby interacting more efficiently with the chitosan polymer chains, causing noncovalent crosslinking [39,40]. This effect of the molecular weight, concentration, and charge on the size of nanogels prepared has been previously described for chitosan–TPP nanogels and various other nanogel compositions [41,42]. The condensation was most effective with the omission of alginate, as evident from the smaller size of the chitosan–TPP nanogels (for both the CT_1_ and CT_2_ compositions). This differs from a report in the literature studying a similar nanogel system by Al-Qadi et al. (2011) [37] that assessed the preparation of nanogels composed of chitosan, hyaluronic acid, and TPP (note that TPP was present in all nanogel compositions). Hyaluronic acid is an anionic polymer similar to alginate, however, in the case of hyaluronic acid, a reduction of particle size was observed with increasing concentrations of the polymer as compared to chitosan. Although the data is not directly comparable, it seems that alginate causes an increase in particle size. The observed differences could be a result of the preparation process, but more likely due to the differences in physicochemical properties of the polymers. Differences in absolute concentrations of the components during nanogel preparation had a minor influence on the size, as evident from the size difference between CT_1_ and CT_2_. A higher concentration led to a reduction in nanogel particle size, but an increase in the PDI. In future studies, modulation of the nanogel particle size could be achieved by changing the alginate content of the nanogels, as shown for hyaluronic acid by Al-Qadi et al. (2011) [37]. The stability data indicated that the different nanogel compositions formed stable nanogels for up to 30 days post-preparation, with no significant changes in the particle size. This is in agreement with other studies on the preparation of chitosan–TPP nanogels [43]. 

The measured zeta potential of the nanogels supports the observed colloidal stability. The zeta potential of the nanogels was positive for all the compositions, as expected, due to the cationic contributions of the chitosan that was added in excess as compared to the anionic components (alginate and TPP). The highly positive surface charge ensures good colloidal stability due to electrostatic repulsion of the nanogels in suspension. The zeta potential of composition CA (+66 mV), containing no TPP, was more positively charged than the other compositions. The lower anionic charge density of alginate compared to TPP likely explains this result, as it leads to a net loss of anionic charge in composition CA as compared to the compositions also containing TPP. The stability studies showed that all the nanogel compositions experienced a significant reduction (*p* < 0.01) in zeta potential over 30 days post-preparation. This reduction in zeta potential may be explained either by the reorganization of the charged molecules within the nanogels or by the presence of a large number of unreacted free anionic polymer molecules in the suspension that may have deposited on the surface of the positively charged nanoparticles, which would negate the positive surface charge over time. This gradual reduction in zeta potential indicates that although the bulk mixed nanogels seem colloidally stable from the particle size measurements, some changes must have occurred on the particle surface. Increased neutralization of the surface charge (below +30 mV) has been demonstrated to induce the collapse of expanded polyelectrolyte chains—which are responsible for the steric repulsion between individual nanogels—leading to their aggregation [44]. This indicates the need for further stabilization of these nanogels, for instance, through formation of NEMs. Although the particle size stability of chitosan–TPP nanogels is well investigated in literature, no reports on the stability of their surface charge was found.

Having characterized the nanogels, NEMs were prepared from the different compositions using two different matrix excipients, trehalose and mannitol. These excipients were selected as they are commonly used for NEM preparation, especially for pulmonary drug delivery purposes. Trehalose is usually obtained in the amorphous form, whereas mannitol is crystalline. The solid-state form of the excipient has been shown to have an impact on the stabilization of nanoparticles in NEMS [45] as well as in spray-dried solid dispersions, where an amorphous matrix is often preferred [46]. The MMADs of CT_2_ and CA NEMs spray-dried with trehalose were 30%–40% greater than NEMs prepared with mannitol. In the case of CT_1_, NEMs with mannitol were 10% larger; for the CTA composition, no difference was observed. The size range of the different NEM powders was between 6 and 10 µm, making the NEMs slightly too large for efficient aerosol deposition to the deep lung if intended for pulmonary drug delivery [12]. However, the particle size could be adequate for other delivery routes and was thus not further considered. 

A great difference in the moisture content of the NEMs was observed between compositions with trehalose or mannitol matrix. The trehalose matrix showed a higher moisture content of 6–12 wt % depending on the embedded nanogels, compared to 3–4 wt % observed for mannitol. X-ray powder diffraction patterns (Figure 4) revealed that trehalose was in a partially amorphous form after spray-drying. This explains the higher moisture content found in the trehalose matrix as compared to mannitol. The NEMs with CA composition had higher moisture content (12 wt %) compared to the other compositions (6–8 wt %), which can likely be explained by higher water retention in the internal structure of CA nanogels, as evident from their larger size (540 nm). Mannitol generally has a moisture content <1%, however, in the NEM samples the moisture content was higher [47], indicating an incomplete dehydration of the nanogels when incorporated in the dry powder NEMs.

Finally, the redispersibility of the nanogels from the NEM powders in an aqueous solution was investigated. Redispersibility is an important characteristic of the NEMs following administration of the dry powder for the nanogels to successfully fulfill their function as drug delivery vehicles in vivo. In the case of the mannitol-based NEMs, no successful redispersion was achieved independent of the nanogel composition. This indicates that mannitol is not a suitable excipient for NEMs embedding nanogel, and is in contradiction to the data reported by Grenha et al. (2005) [36]. In this paper, successful redispersion of chitosan-based nanogels from mannitol-based NEMs was shown, however, the redispersion was allowed to occur over a much longer time frame (90 min) as compared to this work (10 min). In contrast to mannitol, trehalose showed adequate redispersibility of the nanogels independent of their composition. The relative increase in size before and after redispersion for individual nanogels composed of chitosan and TPP was more pronounced (CT_1_ 74% and CT_2_ 76%) than for nanogels that contained alginate in their composition. The CTA and CA nanogels had a relative increase in size of 16% and 46%, respectively. This indicates that alginate improved the redispersibility of the nanogels from the trehalose-based NEMs. This could be explained by the improved ability of the nanogels to rehydrate. However, the mechanism behind this rehydration process should be further elucidated, as it could provide insight into further improvement of NEM redispersibility. 

## 5. Conclusions

This study shows that nanogels composed of different compositions of chitosan, alginate, and TPP can be successfully formulated into NEMs, using a mannitol or trehalose matrix. Trehalose seems to be the preferred matrix excipient, as it facilitated good protection of the nanogel stability (measured by size after redispersion) in the dry NEMs. The trehalose NEMs could be rapidly redispersed into nanogels in an aqueous solution independent of the assessed nanogel composition, without a major size increase. Mannitol has been reported in literature as a suitable excipient for redispersion of chitosan-based nanogels from NEMs. However, for the compositions assessed in this work, performance of crystalline mannitol was inferior to the trehalose with a more amorphous nature. In conclusion, this work highlights the impact of matrix excipient selection and related solid-state properties of the matrix on the redispersibility of chitosan-based nanogels. 

## Figures and Tables

**Table 1:** Different nanogel compositions.

Sample Abbreviation	Chitosan Concentration (mg/mL)	Chitosan:(Alginate + TPP) Ratio (*w*/*w*)	TPP Concentration (mg/mL)	Alginate Concentration (mg/mL)
CT_1_	1	3:1	1.5	-
CT_2_	0.5	3:1	0.75	-
CTA	0.5	3:1	0.60	0.15
CA	0.5	3:1	-	0.75

**Table 2:** Effect of inlet temperatures on resulting (chitosan, sodium tri-penta phosphate (TPP) and alginate (sample CTA)) nano-embedded microparticle (NEM) properties. NEMs were spray-dried at different inlet temperatures. Particle size was measured after redispersion. MMAD: mass median aerodynamic diameter.

Inlet Temperature (°C)	Moisture Content (%)	Particle Size (nm)		MMAD (µm)
		Before Spray-Drying	After Re-Dispersion	
100	11.1	233 ± 0.04	372 ± 0.03	7.92 ± 0.06
120	10.5	233 ± 0.04	236 ± 0.09	7.38 ± 0.10
150	9.7	233 ± 0.04	328 ± 0.02	7.61 ± 0.08
190	10.5	233 ± 0.04	315 ± 0.09	8.06 ± 0.06

**Table 3:** Mannitol-based NEM properties depending on the nanogel composition.

Samples	Moisture Content (%)	Particle Size (nm)		MMAD (µm)
		Before Spray-Drying	After Re-Dispersion	
CT_1_	4.3	230 ± 0.07	>2000	8.23 ± 0.08
CT_2_	3.5	230 ± 0.05	>2000	7.54 ± 0.07
CTA	3.4	240 ± 0.07	>2000	6.68 ± 0.05
CA	3.3	540 ± 0.09	>2000	7.00 ± 0.0.4

**Table 4:** Trehalose-based NEM properties depending on the nanogel composition.

Samples	Moisture Content (%)	Particle Size (nm)		MMAD (µm)
		Before Spray-Drying	After Re-Dispersion	
CT_1_	6.0	230 ± 0.07	402 ± 0.05	7.50 ± 0.04
CT_2_	8.3	230 ± 0.05	406 ± 0.04	10.30 ± 0.06
CTA	7.2	240 ± 0.07	280 ± 0.07	6.75 ± 0.05
CA	11.7	540 ± 0.09	790 ± 0.10	8.90 ± 0.06

## Abbreviations

DLS	dynamic light scattering
MMAD	mass median aerodynamic diameter
NEMs	nano-embedded microparticles
PDI	polydispersity
SEM	scanning electron microscopy
TGA	thermogravimetric analysis
TPP	sodium tri-penta phosphate
wt %	weight percent
